# Taxation and economic sophistication: Evidence from OECD countries

**DOI:** 10.1371/journal.pone.0213498

**Published:** 2019-03-20

**Authors:** Athanasios Lapatinas, Alexandra Kyriakou, Antonios Garas

**Affiliations:** 1 European Commission, Joint Research Centre (DG-JRC), Ispra (VA), Italy; 2 Department of Economics, University of Ioannina, Ioannina, Greece; 3 ETH Zürich, Chair of Systems Design, Zürich, Switzerland; Pennsylvania State University University Park: Penn State, UNITED STATES

## Abstract

Taxation policies can explain the differences in countries’ capacity to produce and export more sophisticated products. We develop a theoretical model considering elements from standard models of economic growth to highlight that a country’s productive structure is implied by the appropriate fiscal policy that is necessary for the development of sophisticated products. We show that economies that rely less on capital relative to labor taxation tend to produce more complex products, while countries that rely more heavily on capital relative to labor taxation produce simple products. These relationships remain robust across alternative econometric specifications. Furthermore, we demonstrate the differential effect of a country’s level of economic development on the nexus between the structure of taxation and economic sophistication. We show that the negative impact of capital taxes on economic sophistication becomes stronger for countries that are more developed.

## Introduction

The ability of a country to develop and grow depends on the transformation of its productive structure, as pointed out early on in the development economics literature [1–4]. On this topic, a series of recent works explain economic development and growth as a process of information development, i.e., a process of learning how to produce and export more complex products [5–8]. In other words, the development path of a country lies in its capacity to accumulate the ‘knowledge’ part of Robert Solow’s growth model, which is required to produce varied and more sophisticated goods [8–10]. However, answers to the questions of (a) why some countries are more developed than others, and (b) how a country can transform its productive structure are still elusive.

The Organization for Economic Co-operation and Development (OECD) argues that long-term differences in the performance of industrial economies arise from the interplay of incentives and ‘knowledge’ [11]. The latter defines the best that can be achieved, while incentives (e.g. fiscal policy) guide the use of ‘knowledge’ and stimulate its expansion, renewal or disappearance. In advanced economies, ‘knowledge’ refers primarily to the supply of human and physical capital, and to the organizational skills required for their use. According to Acemoglu and Zilibotti [12], countries accumulate ‘knowledge’ through the repetition of certain tasks which, in turn, require physical capital in abundance for new machinery, new production systems and components. This implies that accumulation of physical capital is a prerequisite for developing and exporting more sophisticated products. Incentives, on the other hand, originate in public policy and apply on product markets by determining firms’ productivity i.e. the efficiency with which the factors of production are employed. Therefore, the interplay between incentives and factors of production influences the economic complexity of a country through the productivity of its firms. Furthermore, factors of production are strongly interlinked in the process of development [13] and should be bundled when e.g. fiscal policy is designed towards enhancing their accumulation and efficient utilization dynamically [14].

Here, we propose a theoretical model considering elements from standard models of fiscal policy and growth to derive the equation that qualitatively delivers the link between the policy instrument (tax rates) and steady-state sophistication of exported products. According to the predictions of the model, incentives arising from public (tax) policy provide production externalities to firms. Taxation of factors of production changes firms’ productivity. This, in turn, has an impact on the infusion of knowledge and sophistication to new products according to the framework developed by Hausmann et al. [15]. Given that economic complexity is linked to economic growth and that capital determines the accumulation of knowledge which, in turn, leads to the production of more complex goods, tax policies should be used to ensure that firms do not under-invest in their accumulation of capital.

Building upon this intuition we formulate the main hypothesis tested in the paper in the form of a research question: *Do differences in countries’ fiscal policy -everything else being constant across countries (ceteris paribus)- manifest differences in their economic complexity?* To address this question we follow an interdisciplinary approach, and we empirically investigate whether fiscal policy is related to a more sophisticated product space, and whether besides having a direct effect on economic growth [16–23], fiscal policy has an indirect effect on a country’s economic development through the sophistication of its exported products.

Following the relevant literature [6, 7, 24, 25] and using data on countries’ product exports (see Data section), we develop the measure of economic complexity/sophistication (see Methods section). Then, we employ panel data analysis on detailed taxation information for 17 OECD countries for the period 1970-2001, combined with the measure of economic complexity/sophistication. We validate the idea that a greater tax burden on capital leads to the production of less sophisticated products. On the other hand, economies that produce more sophisticated products tend to rely more heavily on labor taxation. Using the ratio of labor to capital taxes, we show that complex productive structures are implied by lower taxes on capital relative to labor. Using fixed-effects two-stage least squares/instrumental variables (FE 2SLS/IV) estimation, we account for the potential reverse causation between taxation policies and economic complexity that may generate an endogeneity problem in the relationship under consideration (see Econometric model section). Our results are backed by several different specifications and sensitivity analyses (see Results section).

In subsection “The impact of economic development on the nexus between taxation and economic sophistication”, we investigate the potential differential effect of economic development on the nexus between the structure of taxation and economic sophistication. We find that the negative effect of capital taxes on economic sophistication is further reinforced by the level of economic development.

Finally, in the last section we discuss the implications of our findings and put forward some concluding remarks.

## Fiscal policy as a determinant of economic complexity

In this section we model the hypothesis to be tested building on a model similar to the one developed by Lapatinas and Litina [26], considering elements derived from standard models of fiscal policy and growth. To study the relationship between fiscal policy and product-sophistication, we first construct the measure of economic sophistication (*EXPY*) using the framework developed by Hausmann et al. [15]. This index captures the productivity level associated with a country’s export i.e. it is a proxy for the most productive set of products the country can produce at a given time. In order to calculate the *EXPY* index goods are ranked according to the income levels of the countries that export them. Products exported by prosperous countries are ranked higher than products exported by poor countries. The aggregation of these product-level calculations leads to the country-wide indexes of economic sophistication.

### Economic complexity

Assuming that *j* denotes the country and *k* the product, the total exports of product *k* from country *j* equal:

$$\begin{array}{c} X_{j}=\sum_{k}x_{jk} \end{array}$$ (1)

If (*y*/*l*)_*j*_ denotes the per-capita GDP of country *j*, the productivity level associated with product *k* equals the weighted average of per capita GDPs, where the weights represent the *Revealed Comparative Advantage* (RCA) of each country for that product:

$$\begin{array}{c} PRODY_{k}=\sum_{j}\frac{\left(x_{jk}/X_{j}\right)}{\sum_{j}\left(x_{jk}/X_{j}\right)}{\left(y/l\right)}_{j} \end{array}$$ (2)

Then, the *PRODY* metric can be used to compute the productivity level associated with country *i*’s economic sophistication, *EXPY*_*i*_, as the average income and productivity level associated with all products exported by a country. It is computed as the weighted average of all relevant *PRODY*s, where the weights represent the share of the relevant product in the country’s export basket:

$$\begin{array}{c} EXPY_{i}=\sum_{k}\frac{x_{ik}}{X_{i}}PRODY_{k}=\sum_{k}\sum_{j}\frac{\left(x_{ik}/X_{i}\right)\left(x_{jk}/X_{j}\right)}{\sum_{j}(x_{jk}/X_{j})}{\left(y/l\right)}_{j} \end{array}$$ (3)

Having derived the equation for the economic sophistication index, *EXPY*_*i*_, we are now going to link this index with the tax rates. Following the extremely rich theoretical literature on fiscal policy and economic growth [27–36] we use here a variant of Barro’s [37] well-known model (Angelopoulos et al. [21], develop a similar model), and we assume discrete time, infinite horizon and perfect foresight.

### Fiscal policy and productivity

#### Households

The representative household maximizes its intertemporal utility:

$$\begin{array}{c} \sum_{t=0}^{\infty }\beta^{t}u\left(c_{t}\right) \end{array}$$ (4)

where *c*_*t*_ is private consumption at time *t* and 0 < *β* < 1 is the discount rate. We follow Angelopoulos et al. [21] and for simplicity we use a logarithmic utility function:

$$\begin{array}{c} u\left(c_{t}\right)=\log c_{t} \end{array}$$ (5)

At each time t, the household rents its predetermined capital, *k*_*t*_, to the firm and receives *r*_*t*_*k*_*t*_, (*r*_*t*_ is the return to capital). It also supplies labor services, *l*_*t*_, and receives labor income *w*_*t*_*l*_*t*_. Further, it receives the profits of its firms, *π*_*t*_. Its budget constraint then is:

$$\begin{array}{c} k_{t+1}+c_{t}=\left(1-\theta_{t}\right)\left(r_{t}k_{t}+w_{t}l_{t}+\pi_{t}\right) \end{array}$$ (6)

where *k*_*t*+1_ is the end-of-period capital stock and 0 < *θ* < 1 is the policy instrument/tax rates (for simplicity we assume full capital depreciation).

The household chooses the paths of *c*_*t*_ and *k*_*t*+1_ in order to maximize its utility given its budget constraint and taking prices and tax policy as given. The first-order conditions of the maximization problem are:

$$\begin{array}{c} \frac{1}{c_{t}}=\beta [\frac{\left(1-\theta_{t+1}\right)r_{t+1}}{c_{t+1}}] \end{array}$$ (7)

and household’s budget constraint, Eq (6).

#### Firms

The representative firm chooses *k*_*t*_ and *l*_*t*_ (taking prices as given) in order to maximize its profits:

$$\begin{array}{c} \pi_{t}\equiv Ak_{t}^{\alpha }l_{t}^{1-\alpha }h_{t}^{\alpha }-r_{t}k_{t}-w_{t}l_{t} \end{array}$$ (8)

where $Ak_{t}^{\alpha }l_{t}^{1-\alpha }h_{t}^{\alpha }=y_{t}$ is firm’s production function. We assume that firm’s technology is represented by a Cobb-Douglas production function which is a form widely used in the economics literature (see Barro and Sala-i-Martin [27], chapter 4). Following the relevant literature (see e.g. Barro [37]) we assume that public services at time *t*, *h*_*t*_, provide production externalities to firms; *A* > 0 is the level of technology and 0 < *α* < 1. The static first order conditions of firm’s maximization problem are:

$$\begin{array}{c} r_{t}=\frac{\alpha y_{t}}{k_{t}} \end{array}$$ (9)

$$\begin{array}{c} w_{t}=\frac{\left(1-\alpha \right)y_{t}}{l_{t}} \end{array}$$ (10)

#### Government

The government runs a balanced budget by taxing the household’s income at a rate 0 < *θ* < 1:

$$\begin{array}{c} h_{t}=\theta_{t}\left(r_{t}k_{t}+w_{t}l_{t}+\pi_{t}\right) \end{array}$$ (11)

#### Competitive decentralized equilibrium

Given the path of the policy instrument (tax rates), ${\left\{\theta_{t}\right\}}_{t=0}^{\infty }$, the equilibrium is defined to be the sequence of allocations ${\left\{c_{t},y_{t},k_{t+1},l_{t}\right\}}_{t=0}^{\infty }$, and prices ${\left\{r_{t},w_{t}\right\}}_{t=0}^{\infty }$ such that: economic agents, households and firms, maximize their utility and profits respectively, by taking prices, tax rates and public services as given, all budget constraints are satisfied and all markets clear. Then, using Eq (11) and *r*_*t*_*k*_*t*_ + *w*_*t*_*l*_*t*_ + *π*_*t*_ = *y*_*t*_ with *π*_*t*_ = 0, the productivity of the economy in the steady-state is given by:

$$\begin{array}{c} \frac{y}{l}=A^{\frac{1}{1-\alpha }}k^{\frac{\alpha }{1-\alpha }}\theta^{\frac{\alpha }{1-\alpha }} \end{array}$$ (12)

### Economic complexity and fiscal policy in the steady-state

Substituting Eq (12) in Eq (3) we get:

$$\begin{array}{c} EXPY_{i}=\sum_{k}\sum_{j}\frac{\left(x_{ik}/X_{i}\right)\left(x_{jk}/X_{j}\right)}{\sum_{j}(x_{jk}/X_{j})}{\left(y/l\right)}_{j}=\sum_{k}\sum_{j}\frac{\left(x_{ik}/X_{i}\right)\left(x_{jk}/X_{j}\right)}{\sum_{j}(x_{jk}/X_{j})}A_{j}^{\frac{1}{1-\alpha }}k_{j}^{\frac{\alpha }{1-\alpha }}\theta_{j}^{\frac{\alpha }{1-\alpha }} \end{array}$$ (13)

This is the equation that qualitatively delivers the link between the policy instrument (tax rates) and steady-state sophistication of exported products. Building upon this intuition we formulate the main hypothesis to be tested in the paper. We thus *empirically test the hypothesis that two countries that differ in their fiscal policy (tax rates)—everything else being constant across countries (ceteris paribus)—manifest differences in economic sophistication. That is, if one of the two countries moves for example to higher capital tax rate relative to labour tax rate, in the steady-state, economic sophistication would be lower in the country with the higher ratio of capital tax to labour tax*.

The following sections describe analytically the data and formally set the equation to be tested which is augmented by additional controls that correlate with the measure of economic complexity.

## Data

### Dataset 1: ECFIN tax rates—OECD panel data

The main explanatory variables are the effective and implicit tax rates on labor and capital provided by Martinez-Mongay [38] and the EU Commission’s department for Economic and Financial Affairs (ECFIN), which are available for 17 OECD countries through the period of 1970-2001. These tax rates are calculated as the ratios between the tax revenues from particular taxes and the corresponding tax bases obtained from national accounts. Specifically, the effective labor tax is defined as the ratio of non-wage labor costs and labor income tax revenues to gross wages. The implicit labor tax rate is the effective tax on employed labor after excluding the taxation of self-employed labor income. Labor taxes include total social security contributions, taxes on payrolls and the workforce, social security contributions paid by employers, as well as taxes on wages and salaries. The capital tax rate includes personal capital income taxes, corporate income taxes and property taxes, where the depreciation is included in the capital tax base. The effective capital tax excludes the imputed wage income of the self-employed, whereas the implicit capital tax incorporates this item as capital income.

### Dataset 2: Economic sophistication

We measure countries’ economic sophistication using the Economic Complexity Index (*ECI*). The *ECI* measures the diversity and sophistication of a country’s export structure, estimated from data connecting countries to the products they export. It is made freely available by MIT’s Observatory of Economic Complexity (http://atlas.media.mit.edu). The index is calculated by applying the methodology described in Hausmann et al. [7] to the folowing datasets that provide details about the products exported by each country: (a) the dataset compiled by Feenstra et al. [39] for the years 1962 to 2000 and (b) the U.N. Comtrade dataset from 2001 to 2012 (https://comtrade.un.org) (a brief description of this methodology is discussed in the Methods section). In short, the *ECI* captures information about an economy’s level of development that is different from what is captured by GDP growth or GDP per capita, for example.

## Methods

To calculate the measure of economic sophistication used in this work, we rely on the methodology described in Hausmann et al. [7]. In short, let us assume that we have trade information for *l* number of countries and *k* products. With this information we can fill an (*l* × *k*) exports matrix **E**, so that matrix element *E*_*ij*_ will be equal to the monetary value country *i* gains by exporting product *j*. Of course, if country *i* does not export product *j*, then *E*_*ij*_ = 0. From this matrix it is easy to calculate the ratio between the share of a given product in a country’s exports and the share of this product in the total global exports. This ratio is called the *Revealed Comparative Advantage* (RCA) [40], and is given by

$$\begin{array}{c} {\text{RCA}}_{cp}=\frac{X_{cp}/\sum_{p^{\prime }}X_{cp^{\prime }}}{\sum_{c^{\prime }}X_{c^{\prime }p}/\sum_{c^{\prime }p^{\prime }}X_{c^{\prime }p^{\prime }}}, \end{array}$$ (14)

where *X*_*cp*_ is the total value of product *p* exports by country *c*. As previously discussed by others [6, 24, 25], a country has a comparative advantage in a product (in other words, is a competitive exporter of a product) when RCA_cp_ ≥ 1.

Using this threshold value, we obtain the (*l* × *k*) matrix **M**, with matrix elements *M*_*cp*_ = 1 if country *c* has a revealed comparative advantage for product *p*, and zero otherwise. This matrix can be viewed as the incidence matrix of a bipartite network linking countries to products.

From this matrix Hidalgo and Hausmann [6] introduced the *Economic Complexity Index* (*ECI*) as a measure of the production characteristics of different countries. To obtain the *ECI*, we calculate the (*l* × *l*) square matrix $\tilde{M}$. In short, matrix $\tilde{\text{M}}$ provides information about links connecting two countries *c* and *c*′ based on the number of products they both export. The matrix elements ${\tilde{M}}_{cc^{\prime }}$ are computed as

$$\begin{array}{c} {\tilde{M}}_{cc^{\prime }}=\frac{1}{k_{c,0}}\sum_{p}\frac{M_{cp}M_{c^{\prime }p}}{k_{p,0}}, \end{array}$$ (15)

where *k*_*c*,0_ = ∑_*p*_ *M*_*cp*_ measures the diversification of country *c* in terms of the number of different products it exports, and *k*_*p*,0_ = ∑_*c*_ *M*_*cp*_ measures the number of countries that export a certain product *p*. If **K** is the eigenvector of $\tilde{\text{M}}$ associated with the second largest eigenvalue, then according to Hausmann et al. [7] the *ECI* is calculated as

$$\begin{array}{c} \text{ECI}=\frac{K-\langle K\rangle }{\text{std}(K)}. \end{array}$$ (16)

## Econometric model

To study the effect of countries’ tax structures on the sophistication of their productive structures, we use dataset 1 (see Data section). The dataset includes 17 OECD countries and spans the period 1970-2001, for which there is information available on both effective/implicit taxation and economic complexity.

In order to avoid the problem of confounding variables, we follow a fixed-effects 2SLS/IV strategy and regress the baseline specification described by the following equation:

$$\begin{array}{c} {\text{ECI}}_{i,t}=\alpha_{0}+\beta_{1}TaxRate_{i,t}+\beta_{k}controls_{i,t}+\gamma_{i}+\delta_{t}+u_{i,t}. \end{array}$$ (17)

Here, the *ECI* is expressed as a function of the tax policy in country *i* and in period *t*, a set of control variables, country *γ*_*i*_ and time *δ*_*t*_ fixed effects, and a stochastic term *u*_*i*,*t*_. We ran different regressions where the main explanatory variable in Eq (17) was the *capital* *tax* *rate* (effective and implicit), the *labor* *tax* *rate* (effective and implicit) and the ratio of (effective and implicit) labor to capital tax burden. The results of these regressions are summarized in Tables 1–6. In what follows, we discuss in detail the control variables, while explicit definitions, descriptive statistics and sources of all the variables employed are provided in the S1 Appendix.

**Table 1:** **The impact of taxation on economic sophistication:** Effective labor tax rate.

	(1)	(2)	(3)	(4)	(5)	(6)
*First stage results*						
Labor tax competition	-0.248***	-0.262***	-0.257***	-0.274***	-0.275***	-0.279***
*Second stage results*						
Effective labor tax	0.080*** (0.015)	0.084*** (0.015)	0.106*** (0.019)	0.095*** (0.017)	0.094*** (0.017)	0.085*** (0.017)
GDP per capita	0.000*** (0.000)	0.000*** (0.000)	0.000*** (0.000)	0.000*** (0.000)	0.000* (0.000)	0.000* (0.000)
population	0.076*** (0.023)	0.064*** (0.024)	0.091*** (0.030)	0.067** (0.030)	0.080** (0.032)	0.061** (0.030)
government expenditure	-0.064*** (0.012)	-0.055*** (0.012)	-0.080*** (0.016)	-0.072*** (0.014)	-0.070*** (0.014)	-0.069*** (0.014)
political globalization	-0.007*** (0.002)	-0.012*** (0.003)	-0.015*** (0.003)	-0.015*** (0.003)	-0.016*** (0.003)	-0.016*** (0.003)
economic globalization	0.001 (0.003)	-0.001 (0.004)	-0.001 (0.004)	-0.002 (0.004)	-0.004 (0.004)	-0.002 (0.004)
democracy		0.021 (0.054)	-0.023 (0.067)	-0.234** (0.096)	-0.165 (0.102)	-0.140 (0.104)
urban			0.029*** (0.009)	0.026*** (0.008)	0.024*** (0.008)	0.028*** (0.008)
corruption				-0.968*** (0.283)	-0.916*** (0.306)	-1.054*** (0.309)
pricexp					2.582*** (0.412)	2.737*** (0.414)
education						-0.000 (0.000)
Time Dummies	Yes	Yes	Yes	Yes	Yes	Yes
Observations	496	434	434	434	434	394
F-statistic	33.11	34.63	34.03	35.02	34.49	33.18
Weak-id	42.43	38.13	33.77	37.07	38.05	37.11
LM-weakid	35.45	33.92	28.18	28.97	28.37	26.67

*Notes*. Estimation method: FE 2SLS/IV; Dependent variable: ECI; The table presents estimated coefficients and robust standard errors in parentheses. All regressions are estimated with time dummies. F-statistic tests the relevance of instruments (whether all excluded instruments are significantly different from zero). Weak-id gives the Cragg-Donald F-statistic for weak identification. LM-weakid gives the Kleibergen-Paap Wald test of weak identification. The *, ** and *** marks denote statistical significance at the 10%, 5% and 1% respectively.

**Table 2:** **The impact of taxation on economic sophistication:** Effective capital tax rate.

	(1)	(2)	(3)	(4)	(5)	(6)
*First stage results*						
Capital tax competition	0.235***	0.216***	0.213***	0.212***	0.213***	0.210***
*Second stage results*						
Effective capital tax	-0.028*** (0.011)	-0.032*** (0.012)	-0.042*** (0.013)	-0.042*** (0.012)	-0.044*** (0.012)	-0.040*** (0.011)
GDP per capita	0.000** (0.000)	0.000* (0.000)	0.000 (0.000)	0.000 (0.000)	-0.000 (0.000)	-0.000 (0.000)
population	0.004 (0.032)	0.006 (0.041)	0.018 (0.047)	0.012 (0.042)	0.021 (0.041)	0.060 (0.041)
government expenditure	0.007 (0.007)	0.009 (0.006)	0.004 (0.007)	0.004 (0.007)	0.005 (0.007)	0.008 (0.007)
political globalization	-0.003*** (0.001)	-0.004** (0.002)	-0.004** (0.002)	-0.005** (0.002)	-0.005*** (0.002)	-0.004** (0.002)
economic globalization	0.011*** (0.002)	0.012*** (0.003)	0.015*** (0.003)	0.014*** (0.003)	0.013*** (0.003)	0.012*** (0.003)
democracy		0.057 (0.057)	0.040 (0.065)	-0.069 (0.111)	-0.027 (0.107)	-0.009 (0.093)
urban			0.019*** (0.004)	0.019*** (0.004)	0.018*** (0.004)	0.015*** (0.004)
corruption				-0.497* (0.297)	-0.459 (0.284)	-0.080 (0.284)
pricexp					1.414*** (0.339)	1.665*** (0.396)
education						0.000*** (0.000)
Time Dummies	Yes	Yes	Yes	Yes	Yes	Yes
Observations	496	434	434	434	434	394
F-statistic	12.93	11.10	11.01	10.52	10.44	10.55
Weak-id	17.48	13.60	12.81	12.82	12.81	12.76
LM-weakid	14.20	11.71	11.60	11.30	11.28	11.24

*Notes*. Estimation method: FE 2SLS/IV; Dependent variable: ECI; The table presents estimated coefficients and robust standard errors in parentheses. All regressions are estimated with time dummies. F-statistic tests the relevance of instruments (whether all excluded instruments are significantly different from zero). Weak-id gives the Cragg-Donald F-statistic for weak identification. LM-weakid gives the Kleibergen-Paap Wald test of weak identification. The *, ** and *** marks denote statistical significance at the 10%, 5% and 1% respectively.

**Table 3:** **The impact of taxation on economic sophistication:** Implicit labor tax rate.

	(1)	(2)	(3)	(4)	(5)	(6)
*First stage results*						
Labor tax competition	-0.244***	-0.300***	-0.269***	-0.290***	-0.278***	-0.310***
*Second stage results*						
Implicit labor tax	0.054*** (0.015)	0.050*** (0.013)	0.076*** (0.018)	0.065*** (0.015)	0.071*** (0.016)	0.065*** (0.014)
GDP per capita	0.000*** (0.000)	0.000*** (0.000)	0.000*** (0.000)	0.000*** (0.000)	0.000** (0.000)	0.000* (0.000)
population	0.048* (0.026)	0.019 (0.026)	0.046 (0.033)	0.022 (0.029)	0.043 (0.031)	0.030 (0.029)
government expenditure	-0.045*** (0.013)	-0.031*** (0.011)	-0.060*** (0.016)	-0.052*** (0.014)	-0.054*** (0.014)	-0.055*** (0.014)
political globalization	-0.006*** (0.002)	-0.012*** (0.003)	-0.016*** (0.004)	-0.016*** (0.003)	-0.018*** (0.004)	-0.017*** (0.003)
economic globalization	0.007*** (0.003)	0.005** (0.003)	0.007** (0.003)	0.006* (0.003)	0.003 (0.003)	0.004 (0.003)
democracy		0.019 (0.056)	-0.031 (0.069)	-0.289*** (0.089)	-0.216** (0.092)	-0.191* (0.098)
urban			0.034*** (0.009)	0.030*** (0.008)	0.029*** (0.008)	0.032*** (0.008)
corruption				-1.188*** (0.265)	-1.164*** (0.281)	-1.236*** (0.301)
pricexp					3.055*** (0.564)	3.325*** (0.580)
education						-0.000 (0.000)
Time Dummies	Yes	Yes	Yes	Yes	Yes	Yes
Observations	496	434	434	434	434	394
F-statistic	20.79	27.71	23.43	27.50	24.77	28.87
Weak-id	25.65	30.51	22.65	26.15	25.09	29.18
LM-weakid	23.75	28.92	20.89	23.57	21.32	22.93

*Notes*. Estimation method: FE 2SLS/IV; Dependent variable: ECI; The table presents estimated coefficients and robust standard errors in parentheses. All regressions are estimated with time dummies. F-statistic tests the relevance of instruments (whether all excluded instruments are significantly different from zero). Weak-id gives the Cragg-Donald F-statistic for weak identification. LM-weakid gives the Kleibergen-Paap Wald test of weak identification. The *, ** and *** marks denote statistical significance at the 10%, 5% and 1% respectively.

**Table 4:** **The impact of taxation on economic sophistication:** Implicit capital tax rate.

	(1)	(2)	(3)	(4)	(5)	(6)
*First stage results*						
Capital tax competition	0.198***	0.175***	0.173***	0.170***	0.170***	0.160***
*Second stage results*						
Implicit capital tax	-0.108*** (0.025)	-0.128*** (0.034)	-0.141*** (0.038)	-0.141*** (0.039)	-0.140*** (0.037)	-0.134*** (0.038)
GDP per capita	-0.000 (0.000)	-0.000 (0.000)	-0.000 (0.000)	-0.000 (0.000)	-0.000* (0.000)	-0.000** (0.000)
population	-0.161*** (0.052)	-0.192*** (0.069)	-0.207*** (0.077)	-0.208*** (0.074)	-0.191*** (0.068)	-0.085* (0.047)
government expenditure	0.038** (0.016)	0.028* (0.017)	0.021 (0.018)	0.021 (0.018)	0.023 (0.018)	0.034* (0.018)
political globalization	-0.002 (0.002)	0.003 (0.004)	0.002 (0.005)	0.002 (0.005)	0.001 (0.004)	0.003 (0.004)
economic globalization	0.023*** (0.005)	0.029*** (0.007)	0.033*** (0.008)	0.033*** (0.009)	0.030*** (0.008)	0.025*** (0.007)
democracy		0.265** (0.119)	0.252* (0.129)	0.225 (0.227)	0.306 (0.215)	0.307 (0.195)
urban			0.025*** (0.009)	0.025*** (0.008)	0.022*** (0.008)	0.013* (0.007)
corruption				-0.122 (0.706)	-0.064 (0.647)	0.896 (0.735)
pricexp					3.137*** (0.857)	3.183*** (0.870)
education						0.000*** (0.000)
Time Dummies	Yes	Yes	Yes	Yes	Yes	Yes
Observations	496	434	434	434	434	394
F-statistic	17.66	14.24	13.98	12.76	13.48	12.38
Weak-id	21.15	15.21	14.46	13.85	14.15	12.80
LM-weakid	15.08	12.40	12.00	10.97	11.47	10.54

*Notes*. Estimation method: FE 2SLS/IV; Dependent variable: ECI; The table presents estimated coefficients and robust standard errors in parentheses. All regressions are estimated with time dummies. F-statistic tests the relevance of instruments (whether all excluded instruments are significantly different from zero). Weak-id gives the Cragg-Donald F-statistic for weak identification. LM-weakid gives the Kleibergen-Paap Wald test of weak identification. The *, ** and *** marks denote statistical significance at the 10%, 5% and 1% respectively.

**Table 5:** **The impact of taxation on economic sophistication:** Ratio of (effective) labor to (effective) capital tax rate.

	(1)	(2)	(3)	(4)	(5)	(6)
*First stage results*						
Ratio L/K tax competition	0.272***	0.239***	0.243***	0.237***	0.243***	0.227***
*Second stage results*						
Ratio (effective) L/K tax	0.718*** (0.142)	0.828*** (0.183)	0.871*** (0.198)	0.858*** (0.204)	0.805*** (0.186)	0.792*** (0.207)
GDP per capita	0.000** (0.000)	0.000 (0.000)	0.000 (0.000)	0.000 (0.000)	-0.000 (0.000)	-0.000 (0.000)
population	0.009 (0.048)	0.038 (0.059)	0.042 (0.063)	0.039 (0.060)	0.045 (0.055)	0.097 (0.062)
government expenditure	-0.010 (0.009)	-0.026** (0.012)	-0.033*** (0.011)	-0.032*** (0.011)	-0.029*** (0.010)	-0.027** (0.011)
political globalization	-0.007*** (0.002)	0.001 (0.003)	0.001 (0.003)	0.001 (0.003)	-0.001 (0.003)	0.001 (0.003)
economic globalization	0.015*** (0.003)	0.018*** (0.004)	0.019*** (0.005)	0.019*** (0.005)	0.016*** (0.005)	0.016*** (0.005)
democracy		-0.052 (0.083)	-0.080 (0.092)	-0.123 (0.145)	-0.071 (0.128)	0.008 (0.124)
urban			0.015* (0.009)	0.015* (0.008)	0.014* (0.008)	0.008 (0.008)
corruption				-0.204 (0.419)	-0.199 (0.362)	0.288 (0.410)
pricexp					2.113*** (0.418)	2.286*** (0.462)
education						0.000*** (0.000)
Time Dummies	Yes	Yes	Yes	Yes	Yes	Yes
Observations	496	434	434	434	434	394
F-statistic	20.41	16.47	16.04	14.45	15.11	11.79
Weak-id	40.93	31.54	32.06	29.91	31.62	23.20
LM-weakid	16.46	13.79	13.37	11.86	12.41	9.30

*Notes*. Estimation method: FE 2SLS/IV; Dependent variable: ECI; The table presents estimated coefficients and robust standard errors in parentheses. All regressions are estimated with time dummies. F-statistic tests the relevance of instruments (whether all excluded instruments are significantly different from zero). Weak-id gives the Cragg-Donald F-statistic for weak identification. LM-weakid gives the Kleibergen-Paap Wald test of weak identification. The *, ** and *** marks denote statistical significance at the 10%, 5% and 1% respectively.

**Table 6:** **The impact of taxation on economic sophistication:** Ratio of (implicit) labor to (implicit) capital tax rate.

	(1)	(2)	(3)	(4)	(5)	(6)
*First stage results*						
Ratio L/K tax competition	0.231***	0.182***	0.185***	0.195***	0.212***	0.222***
*Second stage results*						
Ratio (implicit) L/K tax	0.844*** (0.183)	1.086*** (0.304)	1.144*** (0.321)	1.044*** (0.263)	0.923*** (0.208)	0.811*** (0.181)
GDP per capita	0.000*** (0.000)	0.000*** (0.000)	0.000*** (0.000)	0.000*** (0.000)	-0.000 (0.000)	-0.000* (0.000)
population	-0.097** (0.042)	-0.140** (0.069)	-0.145** (0.073)	-0.148*** (0.057)	-0.115*** (0.044)	-0.054 (0.037)
government expenditure	0.027** (0.013)	0.019 (0.016)	0.015 (0.017)	0.013 (0.016)	0.014 (0.013)	0.019 (0.013)
political globalization	-0.001 (0.003)	0.001 (0.006)	0.000 (0.006)	-0.002 (0.005)	-0.004 (0.004)	-0.004 (0.004)
economic globalization	0.020*** (0.005)	0.027*** (0.007)	0.029*** (0.008)	0.025*** (0.006)	0.020*** (0.005)	0.016*** (0.004)
democracy		0.431** (0.192)	0.428** (0.201)	0.122 (0.211)	0.192 (0.170)	0.169 (0.149)
urban			0.016* (0.008)	0.014* (0.007)	0.011* (0.006)	0.008 (0.007)
corruption				-1.224* (0.708)	-1.097* (0.561)	-0.569 (0.483)
pricexp					3.974*** (0.829)	3.933*** (0.796)
education						0.000*** (0.000)
Time Dummies	Yes	Yes	Yes	Yes	Yes	Yes
Observations	496	434	434	434	434	394
F-statistic	17.05	10.17	10.11	12.12	14.54	15.53
Weak-id	24.25	13.46	13.62	14.85	18.60	16.72
LM-weakid	16.46	10.53	10.51	11.85	14.25	13.90

*Notes*. Estimation method: FE 2SLS/IV; Dependent variable: ECI; The table presents estimated coefficients and robust standard errors in parentheses. All regressions are estimated with time dummies. F-statistic tests the relevance of instruments (whether all excluded instruments are significantly different from zero). Weak-id gives the Cragg-Donald F-statistic for weak identification. LM-weakid gives the Kleibergen-Paap Wald test of weak identification. The *, ** and *** marks denote statistical significance at the 10%, 5% and 1% respectively.

### Control variables

Although the *ECI*’s explanatory variable of capital and/or labor taxes accords well with intuition, in order to ensure robust econometric identification, we use a number of control variables in the estimated equations. Since we do not have prior information (there is no previous research) on the determinants of economic complexity, we have decided to employ a core set of country characteristics similar to that employed in the development economics literature (see also [15]). The strategy applied to deal with potentially omitted country-level covariates is to include likely determinants of economic complexity from the World Bank’s World Development Indicators (WDI), in addition to country fixed effects. In our Results section, we discuss our findings and the estimated relationship between the control variables and the *ECI*.

More precisely, following Hausmann et al. [15], we control for the overall level of productivity and wealth in the economy by employing the real GDP per capita measure (denoted as *GDP* *per* *capita*), using data from Penn World Tables 9.0. We also include population growth (annual %), denoted as *population* and population living in urban areas (% of total population), denoted as *urban*. In order to capture differences between countries in terms of human capital accumulation, we employ total enrollment in secondary *education* [41]. We also control for institutional differences between countries by using the final consumption expenditure of the government (% of GDP), denoted as *government* *expenditure*, as well as the *democracy*-dictatorship data from Cheibub et al. [42]. Furthermore, we include data on *political* *globalization*, *economic* *globalization* and political corruption (*corruption*) from the KOF Index of Globalization [43]. Finally, we employ the price of exports index (*pricexp*) from the Penn World Tables 9.0.

In the Robustness checks section, we verify the robustness of our results by employing the following additional control variables: level of shadow economy (*shadow*); population density (*popdensity*, people per sq. km of land area) and population aged 65 and above (*popold*, % of total) instead of population growth; rural population (% of total population) instead of urban population; *trade* measured as % of GDP and net inflows of foreign direct investment, *FDI* (% of GDP) as alternative measures of *economic* *globalization*. We also establish the robustness of our results to the use of alternative measures of institutional quality, replicating our baseline analysis but substituting the Cheibub et al [42] democracy-dictatorship data with the following measures: (a) *polity*2 from the Polity IV project and (b) constraints on the executives (*executive* *constraints*), also from the Polity IV dataset. See the S1 Appendix for all the variables employed, their definitions and sources. A well-known key issue when adding explanatory variables is that they might exhibit collinearity. For example *GDP* *per* *capita* and *trade* might be highly correlated. For this reason, we add one control at a time, and show that our results are robust to this procedure.

Though we are aware that we cannot fully deal with possible omitted country-level covariates, especially when the above set of control variables is not backed by previous theoretical and empirical research, the findings reported in the next sections suggest that it is unlikely that our results are being driven by excluded country-level factors.

### Instrumental variables

To account for the potential reverse causality between economic complexity and taxation, we use a fixed-effects 2SLS/IV approach. We follow Iosifidi and Mylonidis [44] and we instrument tax rates with their respective measures of tax competition for each country. We expect that the effect of tax competition on economic complexity will be distributed through the effect of changes in taxation and that there will be no direct effect of tax competition on economic complexity. Following the relevant literature [44–46], we construct tax competition measures for both capital and labor taxes:

$$\begin{array}{c} {tc}_{-i,t-1}=\sum_{j=1}^{N}w_{i,j}{tc}_{j,t-1} \end{array}$$ (18)

where the tax competition for country (*i*) is defined as the weighted average of the other countries’ (*j*) tax rates at time *t* − 1, with weights *w*_*i*,*j*_ ≥ 0 if *i* ≠ *j* and *w*_*i*,*j*_ = 0 if *i* = *j*. For the weights, we consider the following formula:

$$\begin{array}{c} w_{i,j}=\frac{\ln(pop_{j}+1)/d_{i,j}^{2}}{\sum_{k\neq j}\frac{\ln(pop_{k}+1)}{d_{i,k}^{2}}} \end{array}$$ (19)

where *pop*_*j*_ is the population of country *j*, *d*_*i*,*j*_ is the air travel distance between countries *i* and *j*, and the denominator is the sum of all other *k* countries. This weight ensures that countries with larger populations exert a relatively stronger effect on other countries’ tax policies, compared to countries with smaller populations. Moreover, following the related literature, we assume that as the geographical distance increases, information and transaction costs increase and profits from economic transactions decline [47]. Therefore, this weight also internalizes the fact that governments care more about the tax policies of their neighboring countries than the tax policies of more distant countries [44].

The underidentification tests (UIT) and the F-statistics for the relevance of instruments (whether all excluded instruments are significantly different from zero), provide evidence of the instruments’ validity.

## Results

### The impact of taxation on economic sophistication

Tables 1–6 show the regression results obtained by applying Eq (17) on the data outlined in the Data section. Table 1 (resp. Table 3) depict the impact of effective (resp. implicit) labor tax on economic sophistication. In all columns the *Effective* (resp. *Implicit*) *labor* *tax* variable bears a positive and highly significant coefficient. This result indicates that higher labor tax rates lead to the production of more sophisticated products.

Table 2 (resp. Table 4) presents the impact of effective (resp. implicit) capital tax on economic sophistication. In all columns, the *Effective* (resp. *Implicit*) *capital* *tax* variable bears a negative and highly significant coefficient. This result indicates that lower capital tax rates lead to the production of more sophisticated products.

Finally, in Table 5 (resp. Table 6) the main explanatory variable is the ratio of effective (resp. implicit) labor to effective (resp. implicit) capital tax rate, which captures the tax burden fallen on labor relative to capital. As shown, *ratio* *L*/*K* *tax* has a positive and significant coefficient that remains robust in all alternative specifications. This highlights that economies relying more heavily on labor relative to capital taxation tend to export more diversified and sophisticated products.

Concerning the rest of the explanatory variables, we observe that *GDP* *per* *capita* bears a marginal positive and significant coefficient in most specifications, indicating that richer countries tend to produce more sophisticated products, whereas *government* *expenditure* has a negative and significant coefficient, highlighting the negative effect of governmental involvement in the production process [48]. *Population* growth rate has an ambiguous effect on *ECI*. More specifically, when the effective labor tax is considered as the main independent variable (Table 1), the coefficient is positive, whereas in the implicit capital tax specification (Table 4) it is consistently negative. *Democracy* doesn’t seem to be a statistically significant determinant of economic complexity in a consistent manner. The two variables of globalization, *economic* *globalization* and *political* *globalization*, capture the crucial elements of economic and political integration. These include, the actual level of cross-border direct or portfolio investment and the interaction and cooperation with other countries, which strengthen the institutions of poor countries and increase their competitiveness. The *economic* *globalization* variable also controls for the fact that capital taxes are less progressive in open economies because capital can flee and the tax burden can be shifted to the less mobile factor of production [44, 49, 50]. *Economic* *globalization* has a positive effect on economic complexity. On the other hand, the *political* *globalization* coefficient is negative (when statistically significant). This result, combined with the negative coefficient of the variable measuring the *corruption* of politicians, might be attributed to the fact that political globalization tends to increase the lobbying and favouritism of political elites [44, 51]. This, in turn—and especially when politicians are corrupt—leads to institutions of lower quality and more exclusive economies. As expected, the price level of exports, *pricexp*, bears a positive and statistically significant coefficient: a higher price of exports is associated with higher diversification and sophistication of the products exported. Finally, the effect of *education* on economic sophistication is positive, implying that human capital increases the sophistication of an economy.

### The impact of taxation on economic sophistication: Robustness checks

In Table 7 (resp. Table 8), we test the robustness of our baseline results by investigating whether the impact of effective (resp. implicit) taxation on the sophistication of productive structures survives under additional and/or alternative control measures.

**Table 7:** **The impact of effective taxation on economic sophistication:** Robustness checks.

	(1)	(2)	(3)	(4)	(5)	(6)	(7)	(8)	(9)	(10)	(11)	(12)
Effective labor tax	0.078*** (0.013)		0.080*** (0.014)		0.088*** (0.018)		0.085*** (0.017)		0.093*** (0.023)		0.080*** (0.019)	
Effective capital tax		-0.049*** (0.018)		-0.061** (0.029)		-0.045*** (0.015)		-0.040*** (0.011)		-0.045*** (0.013)		-0.027*** (0.010)
GDP per capita	0.000 (0.000)	-0.000 (0.000)	0.000** (0.000)	-0.000 (0.000)	0.000* (0.000)	-0.000 (0.000)	0.000* (0.000)	-0.000 (0.000)	0.000 (0.000)	-0.000** (0.000)	0.000** (0.000)	-0.000 (0.000)
population	0.040* (0.024)	0.083 (0.056)					0.061** (0.030)	0.060 (0.041)	0.060* (0.032)	0.083* (0.043)	0.082*** (0.030)	0.071** (0.034)
government expenditure	-0.051*** (0.011)	0.005 (0.007)	-0.058*** (0.012)	0.001 (0.008)	-0.079*** (0.017)	0.006 (0.007)	-0.069*** (0.014)	0.008 (0.007)	-0.085*** (0.024)	0.023*** (0.009)	-0.059*** (0.016)	0.017*** (0.006)
political globalization	-0.013*** (0.002)	-0.004* (0.002)	-0.017*** (0.003)	-0.003 (0.003)	-0.018*** (0.003)	-0.004** (0.002)	-0.016*** (0.003)	-0.004** (0.002)	-0.015*** (0.003)	-0.006*** (0.002)	-0.016*** (0.003)	-0.008*** (0.002)
economic globalization	-0.003 (0.004)	0.014*** (0.004)	0.001 (0.004)	0.016** (0.007)	-0.003 (0.005)	0.014*** (0.004)	-0.002 (0.004)	0.012*** (0.003)				
democracy	0.107 (0.099)	-0.069 (0.108)	-0.177* (0.092)	0.054 (0.186)	-0.163 (0.105)	0.001 (0.125)	-0.140 (0.104)	-0.009 (0.093)	-0.149 (0.112)	-0.061 (0.089)	-0.098 (0.100)	-0.060 (0.069)
urban	0.025*** (0.006)	0.015*** (0.004)	0.011 (0.007)	0.019*** (0.006)	0.028*** (0.008)	0.015*** (0.004)			0.031*** (0.009)	0.011** (0.005)	0.025*** (0.007)	0.012*** (0.004)
corruption	-0.678*** (0.221)	-0.064 (0.329)	-1.386*** (0.279)	0.121 (0.578)	-1.213*** (0.305)	-0.119 (0.358)	-1.054*** (0.309)	-0.080 (0.284)	-1.141*** (0.342)	-0.195 (0.277)	-1.037*** (0.281)	-0.433** (0.207)
pricexp	3.119*** (0.444)	1.455*** (0.462)	2.386*** (0.385)	1.491*** (0.493)	2.646*** (0.414)	1.623*** (0.414)	2.737*** (0.414)	1.665*** (0.496)	2.687*** (0.423)	1.806*** (0.429)	2.417*** (0.406)	1.641*** (0.378)
education	-0.000 (0.000)	0.000*** (0.000)	-0.000** (0.000)	0.000** (0.000)	-0.000 (0.000)	0.000*** (0.000)	-0.000 (0.000)	0.000*** (0.000)	-0.000 (0.000)	0.000*** (0.000)	-0.000 (0.000)	0.000*** (0.000)
shadow	0.095*** (0.021)	-0.036 (0.025)										
popdensity			0.010*** (0.002)	-0.004 (0.003)								
popold					0.023 (0.022)	-0.001 (0.009)						
rural							-0.028*** (0.008)	-0.015*** (0.004)				
trade									-0.003 (0.002)	-0.000 (0.001)		
FDI											0.008 (0.005)	0.000 (0.003)
Time Dummies	Yes	Yes	Yes	Yes	Yes	Yes	Yes	Yes	Yes	Yes	Yes	Yes
Observations	394	394	367	367	394	394	394	394	394	394	356	356
F-statistic	48.03	5.455	39.80	3.605	29.83	7.783	33.18	10.55	21.55	9.605	22.57	10.26
Weak-id	49.45	7.398	45.81	3.731	34.72	8.382	37.11	12.76	24.31	11.33	25.52	12.69
LM-weakid	30.38	5.853	33.35	3.983	26.10	7.920	26.67	11.24	19.14	10.79	19.69	12.88

*Notes*. Estimation method: FE 2SLS/IV; Dependent variable: ECI; The table presents estimated coefficients and robust standard errors in parentheses. All regressions are estimated with time dummies. F-statistic tests the relevance of instruments (whether all excluded instruments are significantly different from zero). Weak-id gives the Cragg-Donald F-statistic for weak identification. LM-weakid gives the Kleibergen-Paap Wald test of weak identification. To save space, the first stage results are not included, and are available upon request. The *, ** and *** marks denote statistical significance at the 10%, 5% and 1% respectively.

**Table 8:** **The impact of implicit taxation on economic sophistication:** Robustness checks.

	(1)	(2)	(3)	(4)	(5)	(6)	(7)	(8)	(9)	(10)	(11)	(12)
Implicit labor tax	0.068*** (0.015)		0.072*** (0.015)		0.070*** (0.017)		0.065*** (0.014)		0.056*** (0.017)		0.060*** (0.017)	
Implicit capital tax		-0.319 (0.224)		-0.168*** (0.054)		-0.129*** (0.036)		-0.134*** (0.038)		-0.182*** (0.060)		-0.151*** (0.048)
GDP per capita	0.000 (0.000)	-0.000 (0.000)	0.000** (0.000)	-0.000** (0.000)	0.000* (0.000)	-0.000*** (0.000)	0.000* (0.000)	-0.000** (0.000)	0.000* (0.000)	-0.000*** (0.000)	0.000* (0.000)	-0.000*** (0.000)
population	0.023 (0.026)	-0.111 (0.109)					0.030 (0.029)	-0.085* (0.047)	0.029 (0.028)	-0.059 (0.058)	0.056* (0.029)	-0.034 (0.049)
government expenditure	-0.048*** (0.013)	0.027 (0.038)	-0.053*** (0.014)	0.027 (0.020)	-0.065*** (0.017)	0.033* (0.018)	-0.055*** (0.014)	0.034* (0.018)	-0.042*** (0.016)	0.102** (0.040)	-0.038*** (0.014)	0.065** (0.026)
political globalization	-0.016*** (0.004)	0.010 (0.013)	-0.019*** (0.004)	0.010 (0.007)	-0.020*** (0.004)	0.003 (0.004)	-0.017*** (0.003)	0.003 (0.004)	-0.017*** (0.003)	0.001 (0.005)	-0.019*** (0.004)	-0.001 (0.005)
economic globalization	0.002 (0.003)	0.064 (0.044)	0.007** (0.004)	0.032*** (0.011)	0.003 (0.004)	0.023*** (0.007)	0.004 (0.003)	0.025*** (0.007)				
democracy	-0.019 (0.097)	-0.195 (0.292)	-0.213** (0.098)	0.505* (0.264)	-0.215** (0.100)	0.296* (0.180)	-0.191* (0.098)	0.307 (0.195)	-0.202** (0.093)	0.350 (0.266)	-0.171* (0.096)	0.285 (0.204)
urban	0.032*** (0.008)	0.018 (0.018)	0.017** (0.007)	0.020* (0.011)	0.034*** (0.008)	0.013* (0.007)			0.029*** (0.007)	-0.000 (0.011)	0.028*** (0.007)	0.004 (0.009)
corruption	-1.013*** (0.261)	1.543 (1.982)	-1.612*** (0.334)	1.622 (1.052)	-1.370*** (0.313)	0.972 (0.712)	-1.236*** (0.301)	0.896 (0.735)	-1.206*** (0.308)	1.201 (1.048)	-1.264*** (0.306)	0.690 (0.816)
pricexp	3.708*** (0.639)	2.934* (1.621)	3.162*** (0.568)	3.360*** (1.083)	3.295*** (0.591)	3.089*** (0.860)	3.325*** (0.580)	3.183*** (0.870)	3.201*** (0.577)	4.340*** (1.227)	3.036*** (0.589)	3.376*** (0.980)
education	-0.000 (0.000)	0.000 (0.000)	-0.000** (0.000)	0.000*** (0.000)	-0.000 (0.000)	0.000*** (0.000)	-0.000 (0.000)	0.000*** (0.000)	-0.000 (0.000)	0.000*** (0.000)	-0.000 (0.000)	0.000*** (0.000)
shadow	0.066*** (0.023)	-0.440 (0.329)										
popdensity			0.012*** (0.003)	-0.003 (0.003)								
popold					0.028 (0.022)	0.018 (0.019)						
rural							-0.032*** (0.008)	-0.013* (0.007)				
trade									0.001 (0.002)	0.006** (0.003)		
FDI											0.009* (0.005)	0.001 (0.006)
Time Dummies	Yes	Yes	Yes	Yes	Yes	Yes	Yes	Yes	Yes	Yes	Yes	Yes
Observations	394	394	367	367	394	394	394	394	394	394	356	356
F-statistic	28.69	1.81	28.43	9.324	25.39	12.50	28.87	12.38	22.47	8.099	18.43	9.023
Weak-id	28.59	2.57	30.15	8.693	25.62	13.43	29.18	12.80	23.50	6.961	18.93	8.205
LM-weakid	21.51	1.86	24.04	8.248	21.35	10.39	22.93	10.54	20.12	7.718	16.39	9.018

*Notes* Estimation method: FE 2SLS/IV; Dependent variable: ECI; The table presents estimated coefficients and robust standard errors in parentheses. All regressions are estimated with time dummies. F-statistic tests the relevance of instruments (whether all excluded instruments are significantly different from zero). Weak-id gives the Cragg-Donald F-statistic for weak identification. LM-weakid gives the Kleibergen-Paap Wald test of weak identification. To save space, the first stage results are not included, and are available upon request. The *, ** and *** marks denote statistical significance at the 10%, 5% and 1% respectively.

Columns (1), (3), (5), and (7) (resp. (2), (4), (6), and (8)) start from the benchmark specification with the full set of controls (column (6) of Tables 1–6) for the effective labor (resp. capital) tax rate and introduce additional variables and alternative measures for some of the previous controls. The same pattern is followed for the results in Table 8 regarding the implicit labor (resp. capital) tax. Specifically, in columns (1) and (2), we adopt a measure of the level of shadow economy (*shadow*), which seems to have a positive effect on the *ECI*. In columns (3) and (4), we substitute the population growth variable with population density, measured as the number of people per sq. km of land area, *popdensity*. An alternative measure of population growth, which also captures differences in countries’ demographic characteristics, is employed in columns (5) and (6), namely, the proportion of people aged 65 and above, *popold*. The first measure has a positive coefficient, while the latter is statistically insignificant. In columns (7) and (8), we use *rural* population (% of total) instead of *urban* population, and in contrast, find a negative effect, as was expected. Columns (9) and (10) employ *trade* measured as % of GDP and columns (11) and (12) consider the net inflows of *FDI* as a % of GDP. The last two variables are considered as alternative measures of *economic* *globalization* (KOF index of globalization). Both variables show a positive association with *ECI* in the specification accounting for the implicit tax rates. Adding these controls in our estimations leaves the findings qualitatively intact.

In addition, Table 9 establishes the robustness of our results to the use of alternative measures of institutional quality. In columns (1) and (2) (resp. (5) and (6)) we use the measure of *polity*2 from the Polity IV project for effective (resp. implicit) labor and capital tax, respectively. In columns (3) and (4) (resp. (7) and (8)), we introduce the measure of the constraints on the executives, also obtained from the Polity IV database. In all columns, we have the full set of controls as in the benchmark specification and we simply replace the democracy-dictatorship data from Cheibub et al. [42]. The results remain qualitatively and quantitatively intact.

**Table 9:** **The impact of taxation on economic sophistication:** Alternative institutional measures.

	(1)	(2)	(3)	(4)	(5)	(6)	(7)	(8)
Effective labor tax	0.076*** (0.014)		0.075*** (0.015)					
Effective capital tax		-0.038*** (0.014)		-0.037*** (0.009)				
Implicit labor tax					0.056*** (0.012)		0.049*** (0.011)	
Implicit capital tax						-0.139*** (0.043)		-0.122*** (0.033)
GDP per capita	0.000* (0.000)	-0.000 (0.000)	0.000 (0.000)	-0.000 (0.000)	0.000* (0.000)	-0.000** (0.000)	0.000 (0.000)	-0.000*** (0.000)
population	0.074*** (0.028)	0.062 (0.044)	0.071** (0.033)	0.030 (0.035)	0.046* (0.026)	-0.110* (0.057)	0.042 (0.033)	-0.094* (0.048)
government expenditure	-0.062*** (0.013)	0.004 (0.006)	-0.054*** (0.013)	0.008 (0.006)	-0.047*** (0.012)	0.026 (0.018)	-0.033*** (0.011)	0.031** (0.015)
political globalization	-0.014*** (0.003)	-0.004* (0.002)	-0.013*** (0.003)	-0.004** (0.002)	-0.016*** (0.003)	0.003 (0.004)	-0.012*** (0.003)	0.003 (0.004)
economic globalization	-0.002 (0.004)	0.013*** (0.003)	-0.004 (0.004)	0.010*** (0.003)	0.004 (0.003)	0.030*** (0.009)	-0.001 (0.003)	0.022*** (0.007)
urban	0.031*** (0.007)	0.013*** (0.004)	0.024*** (0.007)	0.015*** (0.004)	0.035*** (0.007)	0.007 (0.009)	0.027*** (0.006)	0.015** (0.007)
corruption	-1.134*** (0.311)	0.085 (0.334)	-1.950*** (0.350)	-0.395 (0.329)	-1.322*** (0.308)	1.189 (0.903)	-2.306*** (0.339)	0.342 (0.834)
pricexp	2.545*** (0.398)	1.549*** (0.405)	2.347*** (0.390)	1.531*** (0.393)	2.967*** (0.537)	3.047*** (0.925)	2.442*** (0.455)	2.862*** (0.878)
education	-0.000 (0.000)	0.000*** (0.000)	-0.000 (0.000)	0.000*** (0.000)	-0.000 (0.000)	0.000*** (0.000)	0.000 (0.000)	0.000*** (0.000)
polity2	-0.029 (0.018)	0.010 (0.016)			-0.037** (0.016)	0.073* (0.040)		
executive constraints			-0.092*** (0.022)	-0.026 (0.019)			-0.125*** (0.021)	0.003 (0.041)
Time Dummies	Yes	Yes	Yes	Yes	Yes	Yes	Yes	Yes
Observations	367	367	388	388	367	367	388	388
F-statistic	37.63	6.52	35.08	15.71	34.62	9.54	33.40	14.05
Weak-id	44.88	8.30	37.78	17.98	36.11	10.25	37.25	13.44
LM-weakid	28.54	7.60	27.22	14.39	26.06	8.59	26.22	11.92

*Notes*. Estimation method: FE 2SLS/IV; Dependent variable: ECI; The table presents estimated coefficients and robust standard errors in parentheses. All regressions are estimated with time dummies. F-statistic tests the relevance of instruments (whether all excluded instruments are significantly different from zero). Weak-id gives the Cragg-Donald F-statistic for weak identification. LM-weakid gives the Kleibergen-Paap Wald test of weak identification. To save space, the first stage results are not included, and are available upon request. The *, ** and *** marks denote statistical significance at the 10%, 5% and 1% respectively.

### The impact of economic development on the nexus between taxation and economic sophistication

In this subsection we highlight the potential differential effect of a country’s level of economic development on the nexus between taxation and economic sophistication. To identify this channel, we introduce the interaction term *TaxRate* * *GDP* *per* *capita* into Eq (17):

$$\begin{array}{c} {\text{ECI}}_{i,t}=\alpha_{0}+\beta_{1}TaxRate_{i,t}+\beta_{2}TaxRate_{i,t}*GDP\;per\;capita_{i,t}+\beta_{k}controls_{i,t}+\gamma_{i}+\delta_{t}+u_{i,t}. \end{array}$$ (20)

The interaction term *TaxRate* * *GDP* *per* *capita* consists of the product between the mean-centered value of *TaxRate*, and the mean-centered value of the economic development measure, *GDPpercapita*. By taking differences from the mean (mean-centered variables), we avoid the potential problem of multicollinearity between the constitutive terms and the interaction term. The estimation method is again fixed-effects 2SLS/IV with time dummies and the same set of controls as in the benchmark equation. Table 10 presents the results for both the effective (labor in column (1) and capital in column (2)) and implicit tax rates (columns (3) and (4), respectively).

**Table 10:** The impact of economic development on the nexus between taxation and economic sophistication.

	(1)	(2)	(3)	(4)
Effective labor tax	0.086*** (0.016)			
Effective labor*GDP	0.000 (0.000)			
Effective capital tax		-0.028*** (0.010)		
Effective capital*GDP		-0.000*** (0.000)		
Implicit labor tax			0.065*** (0.014)	
Implicit labor*GDP			-0.000 (0.000)	
Implicit capital tax				-0.107*** (0.028)
Implicit capital*GDP				-0.000*** (0.000)
GDP per capita	0.000 (0.000)	0.000 (0.000)	0.000* (0.000)	-0.000 (0.000)
population	0.061** (0.030)	0.041 (0.033)	0.030 (0.029)	-0.064* (0.038)
government expenditure	-0.070*** (0.014)	-0.003 (0.006)	-0.054*** (0.013)	0.017 (0.012)
political globalization	-0.016*** (0.003)	-0.005*** (0.002)	-0.017*** (0.003)	0.002 (0.004)
economic globalization	-0.002 (0.004)	0.012*** (0.003)	0.004 (0.003)	0.025*** (0.006)
democracy	-0.142 (0.105)	-0.036 (0.077)	-0.190* (0.098)	0.234 (0.147)
urban	0.027*** (0.008)	0.017*** (0.003)	0.032*** (0.008)	0.018*** (0.006)
corruption	-1.059*** (0.310)	-0.261 (0.230)	-1.233*** (0.300)	0.531 (0.525)
pricexp	2.755*** (0.411)	1.536*** (0.348)	3.315*** (0.569)	2.583*** (0.690)
education	-0.000 (0.000)	0.000*** (0.000)	-0.000 (0.000)	0.000*** (0.000)
Time Dummies	Yes	Yes	Yes	Yes
Observations	394	394	394	394
F-statistic	34.48	11.75	30.26	17.04
Weak-id	37.98	12.80	30.60	16.97
LM-weakid	27.55	12.99	23.97	14.69

Notes. Estimation method: FE 2SLS/IV; Dependent variable: ECI; The table presents estimated coefficients and robust standard errors in parentheses. All regressions are estimated with time dummies. F-statistic tests the relevance of instruments (whether all excluded instruments are significantly different from zero). Weak-id gives the Cragg-Donald F-statistic for weak identification. LM-weakid gives the Kleibergen-Paap Wald test of weak identification. To save space, the first stage results are not included, and are available upon request. The *, ** and *** marks denote statistical significance at the 10%, 5% and 1% respectively.

We observe that in the case of capital taxation (for both effective and implicit tax rates), its negative impact on economic sophistication becomes stronger at higher levels of economic development. Thus, we show that an increase in capital taxation in more developed countries leads to a more rapid decrease in economic sophistication compared to less developed countries. This is an interesting result with important policy implications. It suggests that policy makers should be cautious when following other countries’ decisions to raise capital taxes, as the impact on economic sophistication is dependent on a country’s level of development and may not be directly comparable.

## Conclusions

It is empirically established in the literature that the sophistication of the products exported by a particular country is related to the country’s pattern of diversification and economic growth. However, what structure of incentives might be most appropriate to enhance investment in the production of more sophisticated goods is still an open question. Here, we explore the effect of the interplay between economic and political factors on the process of government spending transfers from activities of lower productivity into activities of higher productivity.

We show that the structure of taxation policies breeds the diversity and sophistication of a country’s productive structure. Our findings provide strong evidence that a country’s economic sophistication is not only implied by the country’s tax policies, but also by the exact tax structure of the capital tax burden vis-à-vis the labor tax burden (and their ratio). In sum, we find that a country’s capital taxation policy has a negative and statistically strong impact on its productive structure. On the other hand, our results suggest a positive and robust effect of (i) labor taxation and (ii) the ratio of labor to capital taxation on economic complexity. Therefore, we conclude that economies that rely less on capital relative to labor taxation tend to produce and export more varied and sophisticated goods.

This finding is robust to a wide range of econometric specifications. Furthermore, placing the spotlight on the differential effect of a country’s level of economic development on the nexus between taxation and the sophistication of products, we find that the negative impact of capital taxes on economic sophistication is reinforced by a country’s level of income.

Our work is based on the pioneering literature that views development and growth as a process of structural transformation of the productive structure [1–4] enhanced by the accumulation of capabilities [12, 14, 52]. To the best of our knowledge this is the first study that establishes a relationship between tax policies and the sophistication of products and contributes this missing link to a series of recent works explaining economic development as a process of learning how to produce and export more sophisticated products [5, 6, 15, 24, 25, 53].

## Supporting information

S1 Appendix Data sources and descriptive statistics. (PDF)
