# Supplementary material for: Taxation and economic sophistication: Evidence from OECD countries
Source: PLoS One. 2019 Mar 20;14(3):e0213498. doi: 10.1371/journal.pone.0213498 (PMC6426228; doi:10.1371/journal.pone.0213498)
Supplement: S1 Appendix — (PDF) [file pone.0213498.s001.pdf]

Supporting information

Taxation and economic sophistication: evidence from OECD countries

Athanasios Lapatinas[1,2*], Alexandra Kyriakou[2], Antonios Garas[3]

1 European Commission, Joint Research Centre (DG-JRC), Via E. Fermi 2749, TP 361, Ispra (VA), I-21027, Italy
2 Department of Economics, University of Ioannina, P.O. Box 1186, 45110 Ioannina, Greece
3 ETH Zürich, Chair of Systems Design, Weinbergstrasse 56/58, 8092 Zürich, Switzerland

* athanasios.lapatinas@ec.europa.eu

Appendix: Data sources and descriptive statistics

**Variable**	**Description**	**Mean**	**Std. dev.**	**Min**	**Max**	**Source**
Effective labor tax	Effective labour tax: the ratio between the tax revenues from particular taxes and the corresponding tax bases obtained from national accounts. It includes the taxation of the imputed wage of self-employed labor.	29.962	10.415	11.1	54.1	Martinez-Mongay [1]
Implicit labor tax	Implicit labor tax: constructed as above but excluding the taxation of the imputed wage of self-employed labor.	32.740	9.849	13.3	54.2	Martinez-Mongay [1]
Effective capital tax	Effective capital tax: taxation of capital income excluding the imputed wage income of self-employed labor and including depreciation in the capital tax base.	20.318	6.419	6.8	38	Martinez-Mongay [1]
Implicit capital tax	Implicit capital tax: constructed as above but including the imputed wage income of self-employed labor as capital income.	20.147	6.563	5.8	31.7	Martinez-Mongay [1]
ECI	Economic Complexity Index: measure of the diversity and sophistication of a country's export structure	1.451	0.535	-0.006	2.625	MIT's Observatory of Economic Complexity `www.atlas.media.mit.edu`
GDP per capita	Expenditure-side real GDP at chained PPPs (in mil. 2011US$)	1083872	2106345	29055.62	$1.34\ 10^7$	Penn World Tables 9.0
population	Population growth (annual %)	0.499	0.461	-0.428	3.800	World Bank Development Indicators
government expenditure	General government final consumption expenditure (% of GDP)	18.68	3.79	9.93	27.63	World Bank Development Indicators
political globalization	Embassies in country, membership in international organizations, participation in UN security council missions, international treaties. Higher values reflect higher political globalization.	84.44	11.46	53.23	97.94	KOF Index of Globalization [3]
economic globalization	Actual flows (trade, foreign direct investment, stocks, portfolio investment, income payments to foreign nationals), restrictions (hidden import barriers, mean tariff rate, taxes on international trade, capital account restrictions). Higher values reflect higher economic globalization.	64.65	16.55	25.58	97.08	KOF Index of Globalization [3]

Variable	Description	Mean	Std. dev.	Min	Max	Source
popold	Population ages 65 and above (% total Population)	13.38	2.31	7.21	18.36	World Bank Development Indicators
popdensity	Population density (people per sq. km of land area)	139.8	126.1	15.14	475.3	World Bank Development Indicators
democracy	Binary measure of democracy and dictatorship	0.964	0.185	0	1	Cheibub et al. [2]
urban	Urban population (% of total)	72.63	11.82	39.20	97.18	World Bank Development Indicators
corruption	Political corruption index. Higher values reflect higher levels of corruption	0.465	0.136	0.165	0.734	KOF Index of Globalization [3]
pricexp	Price level of exports, price level of USA GDPo in 2011=1	0.465	0.136	0.165	0.734	Penn World Tables 9.0
education	Enrollment in secondary education, both sexes (number)	3563205	5328864	208705	$2.31\ 10^7$	World Bank Development Indicators
shadow	Level of the shadow economy	20.384	7.030	8.94	35.86	Ceyhun and Oguz [4]
rural	Rural population (% of total population)	27.37	11.82	2.82	60.8	World Bank Development Indicators
trade	Trade (% of GDP)	60.31	31.51	10.73	175.17	World Bank Development Indicators
FDI	Foreign direct investment, net inflows (% of GDP)	1.50	2.88	-0.18	25.54	World Bank Development Indicators
polity2	Level of democracy: scale ranges from 0-10 where 0 is least democratic and 10 most democratic	9.55	1.27	1.5	10	Polity IV Project [5]
executive constraints	Executive Constraints (Decision Rules)	6.78	1.00	1	7	Polity IV Project [5]

References

1. Martinez-Mongay C, et al. ECFIN's effective tax rates: properties and comparisons with other tax indicators. European Commission, Directorate-General for Economic and Financial Affairs; 2000.
2. Cheibub JA, Gandhi J, Vreeland JR. Democracy and dictatorship revisited. Public choice. 2010;143(1-2):67–101.
3. Dreher A, et al. KOF index of globalization. Zürich: Konjunkturforschungsstelle ETH Zürich. 2006;.
4. Elgin C, Oztunali O, et al. Shadow economies around the world: model based estimates. Bogazici University Department of Economics Working Papers. 2012;5:1–48.
5. Marshall MG, Jaggers K, Gurr TR. Polity IV Project: Political Regime Characteristics and Transitions, 1800-2004 Dataset Users. Manual, Center for Global Policy School of Public Policy, George Mason University. 2004;.
